# Experiences and Opinions of Adults with Type 2 Diabetes Regarding a Self-Regulation-Based eHealth Intervention Targeting Physical Activity and Sedentary Behaviour

**DOI:** 10.3390/ijerph15050954

**Published:** 2018-05-10

**Authors:** Louise Poppe, Geert Crombez, Ilse De Bourdeaudhuij, Celien Van der Mispel, Samyah Shadid, Maïté Verloigne

**Affiliations:** 1 Department of Movement and Sports Sciences, Ghent University, 9000 Gent, Belgium; ilse.debourdeaudhuij@ugent.be (I.D.B.); celien.vandermispel@gmail.com (C.V.d.M.); maite.verloigne@ugent.be (M.V.); 2 Department of Experimental-Clinical and Health Psychology, Faculty of Psychology and Educational Sciences, Ghent University, Henri Dunantlaan 2, 9000 Ghent, Belgium; geert.crombez@ugent.be; 3 Department of Endocrinology, Ghent University Hospital, De Pintelaan 185, 9000 Ghent, Belgium; samyah.shadid@uzgent.be

**Keywords:** type 2 diabetes, eHealth, physical activity, sedentary behaviour, content analysis, interview

## Abstract

*Background:* Online interventions targeting a healthy lifestyle in adults with type 2 diabetes are more effective when informed by behaviour change theories. Although these theories provide guidance in developing the content of an intervention, information regarding how to present this content in an engaging way is often lacking. Consequently, incorporating users’ views in the creation of eHealth interventions has become an important target. *Methods:* Via a qualitative interview study with 21 adults with type 2 diabetes who had completed an online self-regulation-based intervention (‘MyPlan 2.0’), we assessed participants’ opinions regarding the usefulness of the implemented self-regulation techniques, the design of the programme as well as their knowledge regarding physical activity and sedentary behaviour. A directed content analysis was performed to synthesize the interview data. *Results*: Participants experienced difficulties completing the coping planning component. The simple design of the website was considered helpful, and most participants were aware of the beneficial effects of an active lifestyle. *Conclusions*: ‘MyPlan 2.0’ was well-accepted by the majority of participants. However, the coping planning component will need to be adapted. Based on these findings, recommendations on how to tailor eHealth interventions to the population of adults with type 2 diabetes have been formulated.

## 1. Introduction

Type 2 diabetes (T2D) is associated with numerous health complications and health care visits, resulting in high costs for the patient and society [1]. Consequently, the worldwide exponential growth of T2D has become a major issue [1]. Adopting an active lifestyle, i.e., being more physically active and less sedentary, is considered to be vital in the management of this disease [2,3]. Nevertheless, an active lifestyle is not easily adopted by the majority of patients [4]. Thus, there is a need for the development of various and innovative strategies to promote healthy lifestyle choices in this clinical population [5,6].

One strategy is to provide electronic (e-) health interventions. These interventions can reach many individuals in a cost-effective way and are effective in changing behaviour [7]. They may also prove to be a fruitful avenue to reduce the burden of T2D [8]. Indeed, meta-analyses have shown that online interventions result in modest benefits for T2D management, but larger effects were observed when these interventions were grounded in a behaviour change theory [8,9].

Nevertheless, internet-delivered interventions pose some challenges. Quitting is just a mouse-click away. Hence, many eHealth interventions are subject to high levels of attrition undermining their large potential [10]. This challenge is not adequately addressed by behaviour change theories. These theories provide guidance in developing the content of an intervention, but not in presenting the intervention in an engaging way [7]. For example, providing tailored feedback is an important behaviour change technique, but it requires participants to complete long questionnaires, which may result in high levels of attrition [11]. Consequently, identifying the experiences, opinions and preferences of users regarding theory-based interventions has become increasingly important in the eHealth field [12].

The goal of this paper is to explore how users with T2D experience ‘MyPlan 2.0’, a theory-based eHealth intervention targeting physical activity and sedentary behaviour. ‘MyPlan 2.0’ is informed by self-regulation theory and includes several behaviour change techniques, such as providing information and feedback, creating specific action plans and prompting coping planning [13]. These techniques help people to translate vague goals (e.g., “being more physically active”) to specific actions (e.g., “taking a walk for one hour on each Sunday morning”) [13]. To do so, a qualitative interview study was carried-out. Semi-structured interviews were conducted with participants with T2D after completing ‘MyPlan 2.0’. Based on the results, recommendations can be formulated on how to tailor online interventions promoting an active lifestyle to the population of adults with T2D.

## 2. Materials and Methods

### 2.1. Participants

Participants were recruited via the Diabetes Association Flanders, the Ghent University Hospital and—as some patients brought the researchers into contact with other interested patients—snowball sampling. Eligibility criteria were (1) having T2D; (2) being ≥18 years old; (3) Dutch-speaking; and (4) not having participated in earlier studies with ‘MyPlan’. The study was approved by the Committee of Medical Ethics of the Ghent University hospital (B670201629995), and written informed consent was obtained for all participants. Each participant received a reimbursement of 20 euros for their participation in the study.

### 2.2. MyPlan 2.0

‘MyPlan 2.0’ is based on ‘MyPlan 1.0’, a self-regulation-based eHealth intervention aimed at promoting a healthy lifestyle in the general population. Previous research revealed that ‘MyPlan 1.0’ was effective in changing users’ health behaviours, but faced high levels of attrition [14]. By examining and addressing the features causing attrition (e.g., shortening the programme and applying an easier layout), ‘MyPlan 2.0’ was created. ‘MyPlan 2.0’ is not meant to be a fixed programme, but allows for specific adaptations when targeting particular behaviour and/or particular groups. Here, we discuss the further adaptation for adults with T2D.

**Figure 1:**
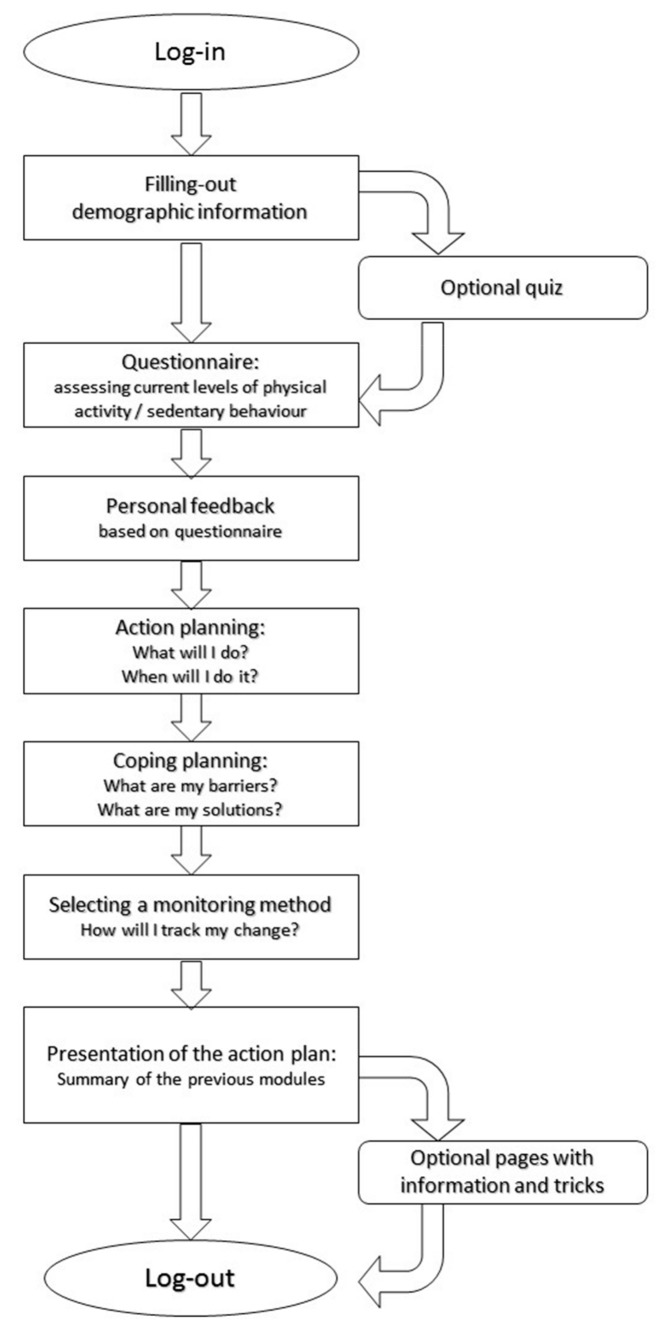
Flow of the first session.

**Figure 2:**
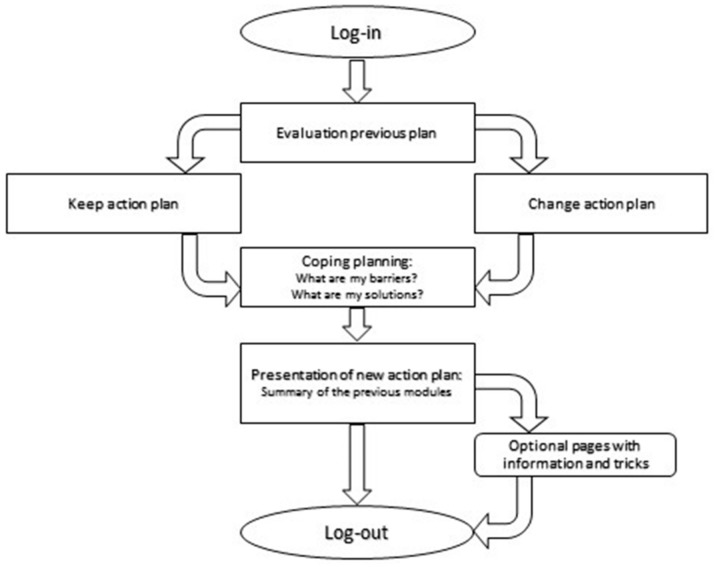
Flow of sessions 2 to 5.

‘MyPlan 2.0’ is a self-regulation-based eHealth intervention (i.e., a website) targeting physical activity and sedentary behaviour. Users of ‘MyPlan 2.0’ can choose between the modules “increasing physical activity” and “decreasing sedentary behaviour”. The website offers five sessions during which users can learn more about the beneficial effects of being less sedentary or more physically active via tips and quizzes (providing knowledge), get feedback on their current levels of physical activity or sedentary behaviour by means of a questionnaire (providing feedback), set their own goals for the coming week (action planning), search solutions for potential barriers (coping planning), think about possible ways to keep track of their behaviour change (monitoring), read optional pages with tips and tricks to become more physically active or less sedentary and evaluate their behaviour change process each week. After an interval of one week, the user receives an email reminding him or her to start the following session. Figure 1 shows the flow of the first session, whereas Figure 2 shows the flow of session 2 to 5.

### 2.3. Procedures

Patients eligible to participate filled out a questionnaire assessing demographic information and were invited to use ‘MyPlan 2.0’ over a period of five weeks (i.e., to go through all sessions of the intervention). If participants forgot to log-in for a next session, they were phoned by a researcher to remind them about the session. After these five weeks, a semi-structured interview took place, which was audio recorded. The interviews took place between January and March 2017, had a duration of approximately 20 min, and were carried out either at the participant’s home, at the university or via telephone depending on each participant’s preference. Supplementary file contains the completed COREQ checklist.

### 2.4. Interview Guide

The interview guide (see Supplementary file) consisted of open-ended questions relating to three main themes. The first theme was “usefulness of the website”, which consisted of several subthemes: (1) personal relevance of the website; (2) stimulating nature of the website; (3) informative value of the website; (4) increased awareness by using the website and (5) recommendations offered by users. The second theme was “design of the website”. This theme consisted of the following subthemes: (1) general perception of the website; (2) user-friendliness; (3) layout and (4) time-efficiency. The third theme was “knowledge”, which relates to the opinions and perceptions of users regarding health behaviours and behaviour change. The themes for the semi-structured interview were based on the results of think aloud interviews with users going through an earlier version of the programme, namely ‘MyPlan 1.0’.

### 2.5. Data-Analysis

A directed content analysis was performed to synthesize the interview data [15]. First, all recordings were transcribed verbatim. Second, a coding scheme was developed, which consisted of the three main themes and nine subthemes from the interview guide and their inclusion and exclusion criteria. Third, two researchers (CVDM and LP) independently coded all interviews using nVivo 11 software (QSR International Pty. Ltd., Melbourne, Australia, Version 11, 2015). Themes not captured by the coding scheme were added to the coding template. A Cohen’s K (weighted for source size) of 0.62 was obtained, indicating fair to good agreement between both coders.

## 3. Results

### 3.1. Participants

Twenty-six participants with T2D volunteered for the study. Five participants dropped out during the study process: two participants never started using the programme, two participants only completed the first session and one participant completed all sessions but could not be reached for the interview. Consequently, there were interviews from 21 participants. One participant only finished four sessions. All other participants completed the whole intervention (i.e., five sessions). Demographic information is shown in Table 1.

### 3.2. Website Usage

In total, participants spent, on average, 48.8 min (SD = 23.1; range = 17–111) on the website. All participants filled out the optional quiz presented during the first session. Table 2 gives an overview of the time that participants spent per session and the number of participants who visited the optional pages at the end of each session.

### 3.3. Interviews

#### 3.3.1. Usefulness of the Website

In response to the question of whether the website provided new information about the importance of increasing physical activity and decreasing sedentary time, many participants responded negatively. Most participants indicated that they were already well-informed by their general practitioner or dietician.

“No, I knew the advantages for your heart, veins and sugar levels.”

(Female, 66 years old)

“I already knew it. Move more often, eat less sweets, those are the basics of diabetes management.”

(Male, 66 years old)

Nevertheless, many participants stated that the website raised their awareness regarding their sedentary behaviour or lack of physical activity, and the fact that they needed to change this behaviour.

“Sometimes I do not think about the fact that I am diabetic but then you receive an e-mail that you need to fill out the website. It awakes the subconscious idea that you need to move more. I feel like they are reminders that keep you awake.”

(Male, 61 years old)

“On each occasion I think about the fact that I should get up and walk a little. I am more aware of this than I used to be.”

(Male, 73 years old)

The questionnaire assessing participants’ current levels of physical activity or sedentary behaviour was considered especially eye-opening. Participants often indicated that they were not aware of the amount of sitting time they accumulated during the day and considered it interesting to gain insight into these patterns.

“I was surprised, I said “ow, I am still sitting a lot”. I often work standing, I iron standing, I prepare meals standing … but still …”

(Female, 67 years old)

“You get confronted with the fact that you do not move very often. And we know it is one of the things you should do as a diabetic. Drink water and move more often. Those are two things that are hard for me and currently lacking.”

(Male, 67 years old)

Almost all participants perceived the website as personally relevant and stated that the website could be used by a broad spectrum of users.

“Yes, yes, absolutely, because being physically active is very important for us!”

(Male, 70)

“I think it fits for every age, even for younger people it would be good.”

(Female, 57)

Participants not considering the website as personally relevant indicated that they were already having an active lifestyle.

“I must say that we already move a lot, so we already did as much as possible.”

(Female, 66)

To explore the extent to which participants adopted the self-regulation-techniques, we asked them whether and how they were helped by specific components of the website. Many stated that creating action plans helped them to actually perform the behaviour because the proposed actions could be easily adopted in everyday life. Some participants stated that they would like to see even more options in the action planning component.

“Yes, that is good! Also because it is not much, well, you do not ask a lot from people. They are small steps that you should take. So each week there are one or two steps and that is achievable. It is not a list of ten things making you say “I need to do all of this!”. No, you do it by yourself, you make your own choices and you get tips and that helps. But you, you do not overwhelm people with it and make it achievable.”

(Male, 61)

“Otherwise I put everything near me: water, the remote, a piece of fruit, it is near me. How many times do I get up then? Not once. Now I leave it here and get it when I need it.”

(Female, 67)

Furthermore, the action planning module motivated participants because they felt they had to keep their promises as they would be evaluated in the next session.

“It was good and I felt a bit obliged, in a friendly way, to get off the couch or off my bed and to do groceries by foot.”

(Male, 69)

“I knew that it (the website) would contact me again, so I had to do something about it!”

(Female, 62)

However, when asking whether participants were helped by reflecting on their barriers and searching for solutions, we obtained mixed answers. Some participants liked the fact that they had to actively reflect on their problems and search for solutions. Nevertheless, most participants felt confused when completing the coping planning component as they found it hard to identify problems and stated that some barriers cannot be easily solved.

“Well, I think it was good that I had to ask myself what is wrong, why are you not coming out of the couch, why are you not walking around, why am I not doing groceries… So, I think that was good.”

(Male, 70)

“Well, there are always barriers, but the solutions are not logic or easy to find.”

(Male, 58)

The website encouraged people to monitor their changes and helped them to evaluate their plan weekly. Many participants liked the fact that they could print the plans as it helped them to remember their promises to the website. Furthermore, participants appreciated that the plan was evaluated at the beginning of each new session.

“I printed it and put it next to my computer. If I forgot it, I could review it.”

(Female, 67)

“I liked the fact that I could evaluate my plans on a weekly basis. I liked this goal-oriented way of working with moments of evaluation.”

(Female, 66)

However, some people believed that monitoring the behaviour change process was superfluous as they could keep it in their mind without additional tools.

“I did not keep track of it, but I kept it in my mind.”

(Male, 70)

#### 3.3.2. Design of the Website

The overall perception of the website was good. Many participants stated that they liked the personal approach. Some participants mentioned that going through the website sometimes still felt like filling out a questionnaire.

“You get the feeling that someone else is taking care of you, individually, you get this feeling.”

(Male, 70)

“I cannot say it is fun, because filling out a questionnaire is not fun.”

(Male, 68)

Generally, participants were satisfied regarding the user-friendliness of the website. They mentioned that the questions were brief and understandable, and that the website was easy to navigate through.

“Yes, it is easy to use and that is nice. You only need to read one thing, not a whole text that you need to go through. These are short things, short questions and it goes well.”

(Male, 73)

“Well yes, I thought it was easy because I told you I do not do anything else (with the computer) and this was very easy that I had to fill out something and go to the next page.”

(Female, 66)

Similarly, participants were also satisfied with the layout of the website. Participants stated that the simple layout helped them to easily navigate through the website without many distractions.

“It is simple and in fact I do not think that is bad, because we are constantly overwhelmed with websites with colours and commercials and other things, I liked it, it was simple but good.”

(Male, 70)

“I really liked this! Yes, yes, very good, simple and it has a positive and playful character … It was not presented as a purely scientific thing … something of which you think “what are they sending me?” No, it is nicely made and remains attractive.”

(Male, 61)

Furthermore, almost all participants stated that they were satisfied with the time-efficiency of the website. Participants liked the short duration of each session as they would be likely to postpone sessions that took more time.

“I think the length was good. It should not be too long, because then you will be less interested of course. Succinctly like they say and that is how it was.”

(Male, 69)

“The time? Oh, that is very doable! You don’t need to spend much time going through the website and then you are finished and you print your plan then it is done. No, no, initially you need to spend a little time on it, but is not worth to talk about that.”

(Male, 73)

#### 3.3.3. Knowledge

Whereas most participants were well aware of the beneficial effects of an active way of living, some questioned whether it was also applicable to them as they did not feel any changes in themselves by being more active.

“Healthy body, healthy mind, it goes together. Because if you feel well, then you will not worry about things that are not good. So if you feel good, by letting your blood circulate by standing up and those things, for example taking the stairs, than you will also feel better on the mental level. That is absolutely true.”

(Male, 61)

“On a mental level it absolutely does (have an effect). On the physical level I have not… I have not really experienced it yet.”

(Male, 73)

#### 3.3.4. Social Support

An additional theme was identified. Several participants mentioned that they went through the website with a family member and experienced social support by doing so.

“I showed it to my husband and told him that I need to move more, because he is of course more physically active than me.”

(Female, 66)

“Yes, sometimes he watched along … I found it interesting. I got a lot of support from that. Yes, by filling it out together. And well … when I had to do something he stimulated me. “It is evening, you need to cycle now” he said. Sometimes I did not feel like doing it, but he said “Come on, you made a promise, you made a deal, you need to do it.””

(Female, 57)

## 4. Discussion

This study assessed the experiences and opinions of adults with T2D regarding a self-regulation-based eHealth intervention targeting physical activity and sedentary behaviour. Investigating whether the target population is ready for an eHealth intervention is an important step before implementing the intervention on a large scale and assessing its effectiveness. Overall, the feedback on ‘MyPlan 2.0’ was positive and highlighted two important issues. First, adults with T2D are a suitable population for eHealth interventions. Second, self-regulation techniques which emphasize patients’ autonomy [13] are appreciated by this population as they feel the need to be in charge of their own behaviour change process. Based on this study, several recommendations on how to further adapt eHealth interventions to adults with T2D can be formulated.

First, although it is tempting to create detailed and elaborated modules for behaviour change techniques, this may come with a cost in terms of time-efficiency. Because the precursor of ‘MyPlan 2.0’, ‘MyPlan 1.0’, was considered too time-consuming, we shortened the programme without omitting any of the implemented behaviour change techniques. This was achieved by providing key messages instead of lengthy texts, creating short questionnaires and making more optional pages. This study shows that many participants appreciated the time-efficiency of ‘MyPlan 2.0’, highlighting the beneficial effect of this endeavour. Similarly, the interview data regarding the user-friendliness and the layout of the website show that our efforts to create an easier and more simple version than ‘MyPlan 1.0’ were appreciated. As the prevalence of T2D peaks in older age [16], users from this population might even favour less complex website designs [17].

Second, the implementation of self-regulation techniques, such as action planning and tailored feedback, was found to be an acceptable method to increase users’ motivation to change their behaviour. Nevertheless, Pall and colleagues found that eHealth interventions in which adults with T2D state specific goals were likely to be ineffective [8]. However, as the authors also note, only five interventions implemented this technique, and of these five interventions, only one intervention gave feedback on patients’ goals. This might be a critical combination. In line with this interpretation, we observed that the action planning component in ‘MyPlan2.0’ prompted participants to live up to their promises because they knew they would be evaluated in the next session. This indicates that eHealth interventions should encourage patients to set specific goals and provide feedback based on their process.

Third, foreseeing future problems and selecting appropriate solutions (i.e., the coping planning technique) has shown to be effective in promoting behaviour change [18]. However, we found that participants experienced difficulties in completing the open answer questions regarding their future problems and solutions, and were not readily convinced of the usefulness of this technique. It may be better to reflect on the barriers of past attempts and then think about barriers to future attempts. Coping planning has been implemented in previous interventions by sending coping strategies via e-mail or SMS to the user [19,20]. Offering potential coping techniques might ease the cognitive process and be a better way of implementing this technique in online interventions targeting adults with T2D. Gradually reducing the pre-built coping plans throughout the sessions might be an option to increase patients’ self-reliance regarding behaviour change.

Finally, several participants wanted to involve their partner when going through the intervention. This is a surprising finding as the feature to send action plans to friends and family was under-used in ‘MyPlan 1.0’, and for that reason, it was deleted in the current version. As social support is a facilitator of behaviour change in adults with T2D [21,22], it might be interesting to explore other ways to involve partners in online interventions targeting an active way of living. For example, based on the patients’ action plan, a page informing the partner regarding how he or she can help the patient to live up to the plan could be created.

To our knowledge, this is the first study to examine the experiences and opinions of adults with T2D regarding a self-regulation-based eHealth intervention. Evidently, our study has some limitations. First, selection bias may hamper the generalizability of our results. Consequently, it might be possible that the patients who were willing to participate in the study differed in some aspects from the general population of adults with type 2 diabetes, such as readiness for behaviour change or computer literacy. Similarly, it is possible that the two participants who only completed the first session and could not be contacted for the interview had different opinions than the participants who were interviewed. These opinions might have given us interesting information about how we could adapt the intervention to individuals who are not yet motivated to complete the programme. Furthermore, 21 patients with T2D might seem a small sample for this study. However, this sample size is in accordance with previous qualitative studies in the eHealth field [12,23,24]. Second, interview data can be distorted by social desirability. Consequently, the participants might have been more positive about the intervention than they actually were. However, as the usage data show, the majority of the participants visited the optional pages of each session indicating a high engagement with the website. Finally, ‘MyPlan 2.0’ consists of five sessions. This number was based on a study of Vandelanotte and colleagues showing that a minimum of five sessions is needed to establish an effect [25]. However, this short intervention period might not be able to establish long-term effects. More research will be needed to assess how users respond to longer versions (i.e., more sessions) of the programme.

## 5. Conclusions

To conclude, we found that adults with T2D are a suitable population for eHealth interventions. The easy and simple design of the programme was appreciated by many participants. Furthermore, this population showed interest in the implemented self-regulation techniques, which were designed to help them to gain autonomy in their behaviour change process. However, the current implementation of the coping planning technique (i.e., searching for possible barriers and solutions) was difficult for the users and should be adapted. Furthermore, several patients liked to involve their partners while going through the intervention. Finally, the effectiveness of ‘MyPlan 2.0’ to increase physical activity and decrease sedentary behaviour in adults with T2D needs to be tested.

## Figures and Tables

**Table 1:** Demographic information.

Demographics	*N* (%)	Mean	SD	Range
Age in years		65.86	5.6	57–81
Women	8 (38.1)			
Level of education Primary school Secondary education College	2 (9.5) 9 (42.9) 10 (47.6)			
Marital status Married Unmarried Divorced Widowhood	15 (71.4) 2 (9.5) 2 (9.5) 2 (9.5)			
Time since diagnosis in months		183.3	155.1	4–480
BMI * in kg/m^2^		30.8	6.1	22.1–42.5

* BMI = Body Mass Index.

**Table 2:** Time spent on the website expressed in minutes.

Session Number	Mean Time Spent (SD; Range)	Number of Participants Visiting Optional Pages (%)
Session 1	22.2 (10.8; 9–46)	15 (71.4)
Session 2	7.1 (4.4; 2–19)	13 (61.9)
Session 3	6.8 (4.3; 2–21)	18 (85.7)
Session 4	6.0 (3.8; 1–15)	13 (61.9)
Session 5	6.5 (6.3; 1–30)	17 (81.0)

## Supplementary Materials

The following are available online at http://www.mdpi.com/1660-4601/15/5/954/s1, Consolidated criteria for reporting qualitative research (COREQ): A 32-item checklist for interviews and focus groups and the interview guide.
