# Supplementary material for: Experiences and Opinions of Adults with Type 2 Diabetes Regarding a Self-Regulation-Based eHealth Intervention Targeting Physical Activity and Sedentary Behaviour
Source: Int J Environ Res Public Health. 2018 May 10;15(5):954. doi: 10.3390/ijerph15050954 (PMC5981993; doi:10.3390/ijerph15050954)
Supplement: Supplementary file 1 [file ijerph-15-00954-s001.pdf]

Supplementary file:

Consolidated criteria for reporting qualitative research (COREQ): A 32-item checklist for interviews and focus groups

Domain 1: Research team and reflexivity

Personal characteristics

1. Interviewer/facilitator. Which author/s conducted the interview or focus group?

LP, CVDM and two Master students (trained by LP and CVDM) conducted the interviews.

2. Credentials. What were the researcher's credentials? E.g. PhD, MD

LP: PhD candidate in Health Sciences and Psychology

CVDM: PhD candidate in Psychology and Health Sciences

IDB: PhD in Psychology

MV: PhD in Physical Education

GC: PhD in Psychology

SS: MD and PhD in Clinical Physiology and Metabolism

3. Occupation. What was their occupation at the time of the study?

LP and CVDM are PhD students performing research; MV is a postdoctoral researcher in health promotion, IDB is full professor in health promotion. GC is full professor in Health Psychology. SS is practicing endocrinologist at the university hospital and lecturer in endocrinology, diabetology and obesity.

4. Gender. Was the researcher male or female?

LP, CVDM, IDB, SS and MV are female researchers, whereas GC is a male researcher.

5. Experience and training. What experience or training did the researcher have?

LP has a Master's degree in Experimental and Theoretical Psychology.

CVDM has a Master's degree in Clinical Psychology.

IDB has a Master's degree in Clinical Psychology and a PhD in Health Psychology

MV has a Master's degree and PhD in Physical Education and Movement Sciences

SS is an MD and has a PhD in Clinical Physiology and Metabolism

GC has a Master's degree in Clinical psychology and a PhD in Psychology

Relationship with participants

6. Relationship established. Was a relationship established prior to study commencement?

No relationship with the participants was established before the commencement of the study.

7. Participant knowledge of the interviewer. What did the participants know about the researcher? e.g. personal goals, reasons for doing the research

The participants knew that the interviewers created 'MyPlan 2.0' and conducted the study in order to ameliorate the programme.

8. Interviewer characteristics. What characteristics were reported about the interviewer/facilitator? e.g. Bias, assumptions, reasons and interests in the research topic

Specific characteristics of the researchers (e.g. training, profession) can always have an influence on data collection and analysis. Nevertheless, we created strict protocols to carry-out the interviews and to analyse the data to minimize bias.

Domain 2: Study design

Theoretical framework

9. Methodological orientation and Theory. What methodological orientation was stated to underpin the study? e.g. grounded theory, discourse analysis, ethnography, phenomenology, content analysis

A directed content analysis was conducted. This type of analysis was considered best suited for our purpose because our coding scheme was based upon previous research with 'MyPlan 1.0'

Participant selection

10. Sampling. How were participants selected? e.g. purposive, convenience, consecutive, snowball

Participants were recruited via the Diabetes Association Flanders, the Ghent University Hospital and snowball sampling.

11. Method of approach. How were participants approached? e.g. face-to-face, telephone, mail, email

Participants were recruited in different ways: face-to-face and via advertisements.

12. Sample size. How many participants were in the study?

Twenty-six participants with T2D volunteered for the study. Five participants dropped out during the study process: 2 participants never started using the programme, 2 participants only completed the first session and one participant completed all sessions but could not be reached for the interview. Consequently, there were interviews from 21 participants.

13. Non-participation. How many people refused to participate or dropped out? Reasons?

Five participants dropped out during the study process: 2 participants never started using the programme, 2 participants only completed the first session and one participant completed all sessions but could not be reached for the interview.

Setting

14. Setting of data collection. Where was the data collected? e.g. home, clinic, workplace

The interviews took place at the research department or via a telephone call. The interviews were audio-recorded with permission of the participants.

15. Presence of non-participants. Was anyone else present besides the participants and researchers?

We asked participants to conduct the interview in a room where they would not be disturbed. However, in some cases we could not prevent that a family member occasionally disturbed the interview.

16. Description of sample. What are the important characteristics of the sample? e.g. demographic data

The demographic information of the sample is provided in table 1 of the manuscript.

Data collection

17. Interview guide. Were questions, prompts, guides provided by the authors? Was it pilot tested?

All questions are provided in additional file 2.

18. Repeat interviews. Were repeat interviews carried out? If yes, how many?

There were no repeat interviews carried out.

19. Audio/visual recording. Did the research use audio or visual recording to collect the data?

All verbalizations were voice-recorded.

20. Field notes. Were field notes made during and/or after the interview or focus group?

No.

21. Duration. What was the duration of the interviews or focus group?

The average duration of an interview was 30 minutes.

22. Data saturation. Was data saturation discussed?

When a text fragment of the interview did not fit any of the predefined categories, a new category was created. Themes that did not contain enough data were not withheld. Coding was done independently by two researchers (CV and LP). A weighted kappa was calculated and showed fair to good inter-rater agreement (weighted kappa: 0.62).

23. Transcripts returned. Were transcripts returned to participants for comment and/or correction?

No.

Domain 3: analysis and findings

Data analysis

24. Number of data coders. How many data coders coded the data?

Two data coders (LP and CVDM) coded the data.

25. Description of the coding tree. Did authors provide a description of the coding tree?

Yes.

26. Derivation of themes. Were themes identified in advance or derived from the data?

The themes were identified in advance. This was based on previous research with "MyPlan 1.0".

27. Software. What software, if applicable, was used to manage the data?

The qualitative data analysis software nVivo 11 (QSR International Pty. Ltd. Version 11, 2015) was used to manage the data.

28. Participant checking. Did participants provide feedback on the findings?

No.

Reporting

29. Quotations presented. Were participant quotations presented to illustrate the themes / findings? Was each quotation identified? e.g. participant number

Yes.

30. Data and findings consistent. Was there consistency between the data presented and the findings?

Yes.

31. Clarity of major themes. Were major themes clearly presented in the findings?

Yes.

32. Clarity of minor themes. Is there a description of diverse cases or discussion of minor themes?

Yes.

The interview guide:

- Which module did you select, less sitting or being more physically active?

Design of the website

Perception of the website

- In general, how do you feel about the website?
- How did you experience using the website?
- To what extent, did you perceive the website as engaging?
- To what extent, did you think using the website was a fun thing to do?
- Could you easily keep your attention to the website?
- Which parts of the website did you like the most?
- Which parts of the website did you like the least?
  - Did you like the questionnaire and its accompanying feedback?
  - Did you like creating an action plan?
  - Did you like searching for barriers and solutions?
  - Did you like reading the tips (e.g. regarding social support)?
  - Did you like monitoring your goal?

User-friendliness

- In general, was the website easy or difficult to use?
- Which parts of the website did you experience as easy?
- Which parts of the website did you experience as difficult?

- How do you think about the user-friendliness of the website?
    - Could you easily find what you needed?
    - Where there moments that you were stuck?
    - Could you easily read all the text?
- Was the questionnaire and it accompanying feedback easy/difficult?
- Did you perceive creating your own action plan as easy/difficult?
- Did you perceive searching for barriers and solutions as easy/difficult?
- Were the tips easy/difficult?
- Did you perceive monitoring your goal as easy/difficult?

Time efficiency

- What do you think about the time you needed to complete the sessions?

Lay-out

- What do you think about the lay-out and the design of the website?

<u>Usefulness of the website</u>

Recommendations

- Do you suggest any adaptations to the website?
- Are there things you would recommend us to change regarding the website?

Stimulating value of the website

- To what extent did the website help you to change your behaviour?
- Which parts of the website helped you the most?
- Which parts of the website helped you the least?
    - Was it helpful to create an action plan?
    - Was it helpful to search for barriers and solutions?
    - Was it helpful to read the tips (e.g. regarding social support)?
    - Wat is helpful to monitor your goal?

Awareness

- To what extent did the website make you more aware?

Personal relevance

- Did you perceive the website as personal relevant?
- Did you perceive the questionnaire and its accompanying feedback as personal relevant?

Informing value of the website

- To what extent did you learn new things by using the website?
- To what extent did you learn new things regarding behaviour change?
- To what extent did you learn new things regarding being less sedentary or more physically active?

Knowledge

- Do you think sitting less/being more physically active has an influence on how feel physically/mentally?
- Do you think sitting less/being more physically active has an influence on your health?
